# Supplementary material for: Remote home monitoring (virtual wards) for confirmed or suspected COVID-19 patients: a rapid systematic review
Source: eClinicalMedicine. 2021 Jun 23;37:100965. doi: 10.1016/j.eclinm.2021.100965 (PMC8219406; doi:10.1016/j.eclinm.2021.100965)
Supplement: Supplementary file 1 [file mmc1.docx]

***Appendix 1.***  Phased search strategies and PICO

COVID-19

AND

“virtual ward” OR “remote monitoring” OR “virtual monitoring” OR “home monitoring” OR “community monitoring” OR “early monitoring”

"COVID-19"[All Fields] OR "severe acute respiratory syndrome coronavirus 2"[All Fields] OR "severe acute respiratory syndrome coronavirus 2"[All Fields] OR "2019-nCoV"[All Fields] OR "SARS-CoV-2"[All Fields] OR (("Wuhan"[All Fields] AND ("coronavirus"[MeSH Terms] OR "coronavirus"[All Fields])) AND 2020[All Fields])

AND

“virtual ward” OR “remote monitoring” OR “virtual monitoring” OR “home monitoring” OR “community monitoring” OR “early monitoring” OR “remote patient monitoring” OR “pre-hospital monitoring” OR “Covidom” OR “My m health” OR “GetWell Loop” [All Fields]

AND

“silent hypoxemia” OR “pulse oximetry” [All Fields]

COVID-19 OR

AND

“virtual ward” OR “remote monitoring” OR “virtual monitoring” OR “home monitoring” OR “community monitoring” OR “early monitoring” OR “pre-hospital monitoring”

AND

“silent hypoxemia” OR “pulse oximetry”

PICO framework

| Population | Suspected or confirmed COVID-19 patients |
| --- | --- |
| Intervention | Remote home monitoring |
| Comparison | N/A |
| Outcome | Virtual length of stay, escalation, emergency department attendance/reattendance, admission/readmission and mortality |

***Appendix 2.*** Characteristics of the included remote home monitoring examples

| **Author** | **Country** | **Type of study and article** | **Type of model** | **Terms** | **Sector** | **Patient population** | **Triage process** | **Recorded patient info** | **Patient reporting tool** | **Patient monitoring tool** | **Outcomes** |
| --- | --- | --- | --- | --- | --- | --- | --- | --- | --- | --- | --- |
| Margolius [4] | USA | Evaluation/ Preprint | Pre-admission | Telehealth services | PC | C19 symptoms | Patient referred to teleconsultation and follow-up call made 24 hours after | ***Data for risk assessment***:  age, sex, race/ethnicity, insurance type, smoking status and clinical variables directly relevant to understanding the social epidemiology of the COVID-19 hotline (symptom protocols, visit disposition, visit diagnoses).  ***Data for monitoring***: change in symptoms (including temperature), basic living needs | Paper-based | Telephone call | (1) emergency room visit likely related to COVID-19 subsequent to hotline telehealth visit, (2) hospitalization due to COVID-19 subsequent to hotline telehealth visit, (3) SARSCoV- 2 PCR test ordered subsequent to telehealth visit, and (4) positive SARS-CoV-2 PCR test subsequent to telehealth visit. |
| Maghrabi*[18] | UK | Description of the service/ Preprint | Step-down ward | Virtual ward | SC | Discharged patients with suspected or confirmed COVID-19 | Patients where there were concerns about oxygenation were discharged with a pulse oximeter and onboarded on to the virtual ward. | ***Data for monitoring:*** symptom improvement, stability or deterioration (including oxygen saturation) | Patients received daily phone calls and asked standardised questions. | Patients referred through an electronic form on the EHR, monitoring data were inputted directly on the EHR and auto-populated a dashboard. | LoS on virtual ward and in hospital, O_2_ requirements, readmission, reattendance, mortality and patient satisfaction. |
| Thornton* [5] | UK | Description of the service in news feature/ Published article | pre-admission and step-down | Virtual ward | SC | Patients presenting at ED with symptoms and admitted patients who needed additional monitoring at the point of discharge | Patient assessed in ED and triaged to virtual ward with pulse oximeter. Patients where there were concerns about oxygenation were discharged with a pulse oximeter and onboarded on to the virtual ward. | ***Data for risk assessment***:  patient-reported data (clinical signs and symptoms, medical history and medications)  ***Data for monitoring***: symptoms, temperature, and their oxygen level. | One example used Online: App (Medopad) + phone calls (phase 1 of the service)  Second example used: Paper-based (patient recorded information at home and reported it to the medical team over the phone) | Medopad app  Phone call with medical team. Patients asked to do stress test and report O_2_ sats level | ED reattendance, admission/readmission, mortality |
| Hutchings* [6] | Australia | Observational study/ Preprint | Pre-admission | Virtual health care, remote patient monitoring | SC | Patients in whom C19 is detected (certain inclusion and exclusion criteria apply) | Patients attend COVID-19 testing clinic, those in whom the virus is detected are referred to the virtual care centre by the local public health unit. The care centre conducts an initial assessment to ascertain suitability for virtual health care – this is done by telephone | ***Data for risk assessment***:  patient-reported data (clinical signs and symptoms, medical history and medications)  ***Data for monitoring***:  vital signs - respiratory rate, oxygen saturation, pulse rate and temperature, assessment of other symptoms and signs of deterioration assessed by video call | Online: Wearable temperature monitor provides continuous temperature monitoring, which feed into a dashboard.  Pulse oximeter readings read directly from the device.  Video consultations used to confirm vital signs collected by wearable devices. | Wearable temperature monitor provides continuous temperature monitoring, which feed into a dashboard. Patients monitored three times a day, including a videoconference twice every 24 hours. Video consultations comprised most contacts with telephone consultations making up the remainder. | Ambulance attendance, ED attendance, ED admission, mortality |
| Kricke [7] | USA | Description of service/ Published article | Pre-admission | Home monitoring, outpatient monitoring, community based virtual care | SC | Patients added to the registry were those with pending/indeterminate/positive COVID-19 test or presumed presence based on clinical criteria. Later began only including those with positive COVID-19 test. | States only nurses from COVID-19 triage phone line, ED staff, and hospital medicine staff were able to add patients to the registry. | ***Data for risk assessment***:  Evaluation of 10 symptoms – used a short questionnaire that captured the main domains of patients’ symptoms and experiences. (symptoms - cough, shortness of breath, sore throat, muscle aches, trouble sleeping, lack of energy, feeling ill, fever, diarrhoea, stomach pain), patients were also asked how many analgesic/antipyretic tablets they are taking, symptoms of others in the household, and for a measured temperature.  ***Data for monitoring***: symptoms and temperature. | Online: Enrolled patients with an electronic health record portal account receive a questionnaire invitation where they evaluate symptoms, those not enrolled in the patient portal (or who do not respond) are called. | They monitor and stratify responses to daily questionnaires, those with concerning symptoms are called. Calls used to evaluate symptoms, provide information and answer questions. | ED referrals (also anecdotal data about being provided education, comfort and getting 911 activation help). |
| Annis*** [8] | USA | Evaluation/ Published article | Pre-admission | Remote patient monitoring, telehealth | SC | Patients with confirmed or suspected COVID-19 | Patients that were enrolled were either screened for COVID-19through virtual care platforms (phone, video, online) or at an ED or urgent care visit and referred. Providers were informed about the programme as a care option. Had a referral order within electronic health records to gather the patients’ required information and they developed a batch process to automate enrolment. Then patients received an email with information on how to activate and begin the programme (optional). | ***Data for risk assessment***:  patient-reported data (clinical signs and symptoms, medical history and medications).  ***Data for monitoring:*** Daily check in questions to monitor/assess symptoms, later updated to include question that assessed pulse oximetry data. | Online: GetWell Loop - daily check in questions for patients to assess their symptoms, patients could also send comments and questions through scrolling newsfeed. Patients could also call the Mhealth triage line for alerts or comments outside 8am-5pm (before they expanded the workforce to include 24/7 virtual care so alerts could be responded to out of hours). | GetWell Loop - symptom monitoring questions were monitored - concerning answers routed to dashboard for action by member of first responder team. Physicians would also text or call patients if an alert or comment was concerning/complicated. | Hospital admissions, ED visits. Patient satisfaction data also collected. |
| O'Keefe [12] | USA | Observational study (retrospective cohort study)/  Preprint | Pre-admission | Telemedicine visits, virtual outpatient management, telephone monitoring | SC | Patients with positive COVID-19 PCR test | Patients with positive COVID-19 PCR test from screening clinics or ED were referred for enrolment in the Virtual Outpatient Management Clinic. For those enrolling in the virtual clinic, risk assessment data were obtained during a scheduled telemedicine appointment. | ***Data for risk assessment***:  patient-reported data (clinical signs and symptoms, medical history and medications).  ***Data for monitoring:***  Reported symptom data (including temperature). | Patients received regular calls | Telephone - patients received regular calls, different levels of observation e.g. frequency of calls and duration, based on assigned risk tier. | Hospitalisation (metric: days to hospitalisation). |
| Ford* [14] | USA | Description of the service/ Published article | Pre-admission and step-down ward | Telehealth remote patient monitoring | SC | Patients with confirmed COVID-19 | Used dedicated registry of COVID-19 patients - populated using the positive diagnostic test as the trigger (as well as with all patients using virtual urgent care for COVID-19 suspicions). All testing submitted through the site was pulled into the registry for potential enrolment in home monitoring as were all positive tests regardless of entry point (drive up, virtual urgent care, ED, inpatient admission or pre-op testing). Nurses could enrol, triage and follow patients - nurses contacted patients who tested positive and offered opportunity to enrol in programme. | ***Data for risk assessment***:  patient-reported data (clinical signs and symptoms, medical history and medications)  ***Data for monitoring:*** Patient reported outcomes (PRO) survey– derived from validated community acquired pneumonia patient questionnaire (five item survey queries changes in patient reported dyspnea), later extended to include pulse oximetry (for select groups inc post hospitalisation) and digital thermometers (app also extended capabilities with Bluetooth pulse oximeters and digital thermometers). | Online: Via patient portal (Epic MyChart electronic health record) or app- nurse managers could choose which to prescribe. | Monitored responses to PRO through portal or app, nurses can reach out by phone if symptoms worsen. | Nurse to patient encounter, referral for physician review, physician call, referral to ED, hospitalisation. |
| Agarwal* [17] | Canada | Observational study (retrospective cross-sectional study)/ Preprint | Pre-admission | Remote home monitoring model, virtual care | PC | Patients with COVID-19 (swab or presumed positive) felt to be high-risk based on age, comorbid  illness and respiratory symptoms. | Patient attended PC and was triaged to low, moderate or high risk using clinical judgement. Follow-up virtual visits were booked with the resident or RN every 1-3 days based on risk. Program aimed to follow patients from  time of referral up to 14 days from symptom onset. | ***Data for risk assessment:*** Demographics, comorbidities, COVID status, risk of transmission, symptoms, oximeter readings, thermometer readings.  ***Data for monitoring:*** symptoms, oximeter readings, thermometer readings. | Paper-based: Telephone calls directed by medical team but patients also  had access to a dedicated on-call service 24-hours a day. Pulse oximeters and thermometers were couriered to patients. | Telephone or  video visits, 7-days a week by an inter-professional, family medicine led team. Data entered into EPIC using a standardized  electronic flowsheet. A dashboard cataloguing each patient in the program with their risk level for deterioration and active care issues was developed to facilitate daily team  huddles. | ED attendance, admission, referral to social worker. |
| Xu [21] | China | Observational study (retrospective cohort study)/ Published article | Pre-admission | Telemedicine system | SC | Confirmed or suspected cases of COVID-19 | Patient attended hospital and was assessed for telemedicine system. The patient was given access to an online telemedicine form and a link to the WeChat app to the patient’s mobile phone or by email. | ***Data for risk assessment:***  Demographics, clinical history, clinical manifestations, lab tests, CT images  ***Data for monitoring:*** changes in symptoms (including temperature). | The patient was required to update their conditions on a daily basis using the telemedicine form. The patient joined a WeChat group to receive information and could set-up a one-to-one chat with the MDT. | Communication through telemedicine form and WeChat group. | ED attendance, admission, mortality, need for ECMO. |
| Medina* [13] | USA | Service description/ Published article | Pre-admission and step-down | Home monitoring, home-based intervention | SC | Confirmed COVID-19 and risk factors: risk factors include age older than 60 or younger than 3, active immunosuppression, active cancer, end-stage renal disease on dialysis, diabetes, hypertension, coronary artery disease, heart failure with reduced ejection fraction, chronic lung disease, HIV/AIDS, and organ transplant. | Patients are enrolled into the home monitoring program after an ambulatory virtual assessment with a clinician, or after hospital discharge for COVID-19. Patients receive an initial phone call with instructions. Patients from the hospital are monitored for 7 days and ambulatory patients for 14 days. | ***Data for risk assessment***:  patient-reported data (clinical signs and symptoms, medical history and medications).  ***Data for monitoring:*** Symptoms, pulse oximetry readings, temperature. | Online: Patient records information on the MyCare Companion app. | Daily monitoring of patients consists of telephonic outreach from a registered nurse or allied health professional and a self-monitoring app (MyCare Companion) that allows for patient-entered data. A pool of nurses and clinicians monitor the EMR registry and flag symptoms that are worsening. After a nursing assessment, a patient may then be escalated for additional care (virtual call or referral to ED). | Time to escalation, ED attendance, admission, mortality. |
| Lam** [20] | Canada | Feasibility study/ Published article | Pre-admission | Virtual care program | SC | Adult patients who tested positive for COVID-19. | Infection Prevention and Control receives positive test result. Patient contacted via phone to onboard. Follow-up was discontinued after signs of clinical improvement. | ***Data for risk assessment:***  Clinical and travel history, symptoms, exposure.  ***Data for monitoring:*** changes in symptoms (including temperature). | Online and paper-based: Patients record data on the Ontario Telemedicine Network virtual care platform, but if they refuse, these data were collected by phone. | Stable patients contacted a minimum of once a week, patients who were deemed to require more frequent follow-up were contacted up to twice a day by telephone. Escalation arranged by the service to ED. | Time from swab collection to first assessment, duration of virtual care, ED attendance, admission, ICU admission, mortality. |
| Grutters* [26] | The Netherlands | Description of service/ Published article | Step-down | Home telemonitoring, remote patient monitoring | SC | Hospitalised patients considered appropriate for discharge with remote monitoring. | When patient’s clinical condition in hospital improved, they were approached for the home monitoring service. | ***Data for risk assessment:***  Clinical and travel history, symptoms, exposure  ***Data for monitoring:***  Symptoms, pulse oximeter and temperature readings. | Online: Patient recorded data on an app on a daily basis. Patients were able to post comments on the app. | Data from the app displayed on a real-time basis on a dashboard. Staff also made calls to help with oxygen therapy queries and for follow-ups. | ICU admission, LoS, reassessment at hospital, readmission, mortality, patient experience, costs. |
| Shah* [16] | USA | Observational study (prospective study)/ Published article | Pre-admission | Home pulse oximetry monitoring | SC | Confirmed or suspected COVID-19 presenting in ED. | Patient discharged from ED with confirmed or suspected COVID-19 and were given a pulse oximeter. Patients were followed-up for 7 days. | ***Data for risk assessment:***  Demographics, medical history, lab tests  ***Data for monitoring:***  Symptoms, pulse oximeter and temperature readings. | Paper-based: Patients recorded measurements and communicated these to staff once a day during a phone call. | Patients were called once a day. | Admission, resting pulse oximeter readings, LoS, ICU admission, time to drop, development of acute respiratory distress syndrome, septic shock, mortality. |
| Morgan** [28] | USA | Description of the service/ Published article | Pre-admission and step-down | Remote monitoring patients | SC | Confirmed or suspected cases. | Patient could be added to the service after testing positive, during an ED visit, a call or telehealth visit with any clinician, or following discharge  from an inpatient admission. | ***Data for risk-assessment:*** symptoms  ***Data for monitoring:*** symptoms | SMS based: patient received twice-daily message and could reply to messages sent by the clinical team. | Patients were monitored by a nurse as information submitted by patients triggered EHR inbox message. | ED attendance, admission, length of stay, escalation. |
| O’Carroll* [15] | Ireland | Description of the service/ Published article | Step-down | Remote monitoring | SC | Confirmed patients deemed suitable for discharge | Patients who could be discharged from hospital as they did not require supplemental oxygen. | ***Data for monitoring:*** symptoms, oxygen saturations (pulse oximetry). | Pulse oximeters were connected to an app. The app sent a prompt to patients to record oxygen saturations 4 times a day. | The app triggered an alert and the medical team contacted the patient and gave instructions on next steps. | Readmissions, length of stay, ICU admission |
| Bell [19] | UK | Description of service/ Published article | Pre-admission | Remote monitoring, rapid follow-up | SC | Confirmed or suspected cases. | Patient discharged from ED and given a pulse oximeter based on pre-established criteria (CPR >50; RR>20; O2 saturation 94 or 95%; exercise desaturation >2%). | ***Data for risk assessment:*** not specified  ***Data for monitoring:*** symptoms, oxygen saturations (pulse oximetry, demographics, | Telephone assessment made 36 hours post ED visit in the first instance and an electronic proforma was used to document the assessments. The frequency of follow-up depended on the level of risk of the patient. | Signs of deterioration identified by the clinical team during calls or initiated by ‘patient-activated’ calls could prompt the need for face to face review. Patients with symptoms post 28 days were referred to a respiratory clinic. | Reattendance (planned and unplanned), admission, referral to respiratory clinic, referral to other secondary care clinic. |
| Gaeta [27] | USA | Description of service/ Published conference abstract | Pre-admission | Remote monitoring, rapid follow-up | SC | Confirmed or suspected cases. | Patient discharged from ED and given a pulse oximeter or PO + oxygen concentrators based on pre-established criteria (RR<22; O2 saturation 90% or above). | ***Data for risk assessment:*** oxygen saturations and RR.  ***Data for monitoring***: symptoms, O2 saturations, HR, RR. | Daily telehealth consultations for seven days. Observations recorded on patient charts. | Not specified | Reattendance, disease course, hospital LoS, ICU requirements, respiratory support, mortality and loss to follow-up. |
| Gordon [37] | USA | Description of service/ Published article | Step-down | Remote patient monitoring | SC | Confirmed or suspected cases. | Patient referred to the service at the time of discharge. The service was for adult patients who did not have comorbid highly symptomatic non-COVID-19 conditions or cognitive barriers to use the devices. | ***Data for risk assessment:*** not specified.  ***Data for monitoring:*** symptoms, O2 sats, temperature. | Patients used an app (MyChart Care Companion) to record observations and abnormal symptoms were flagged and assessed by nurses leading the service. | Symptoms that worsen or no response from patient triggered a message to the the nursing team who would then call the patient. Patient could be referred to doctor to determine if a visit to the ED was required. | Length of stay, readmission, ED attendance. |
| Kodama [25] | USA | Description of service/ Published article | Step-down | Remote patient monitoring | SC | Confirmed or suspected cases, older than 18 years, O2 sats < 92%, confidence using a PO. | Patient was identified at the point of discharge based on pre-established criteria. | ***Data for risk assessment:*** age, O2 sats.  ***Data for monitoring:*** O2 sats, HR, RR, | Patients used an app to input observations and signs were monitored twice a day. | Triggers for escalation appeared on a dashboard monitored by a nurse. The nurse also called patients twice a day. | ED attendance, admission. |
| Nunan [22] | UK | Evaluation/  Published article | Pre-admission and step-down | Virtual monitoring, virtual ward | SC | Confirmed or suspected cases. | Patient was triaged into one of three groups based on O2 sats. Those with O2 sat > 94% could be referred to virtual ward. Some patients referred after discharge and others prior to admission. Patient was given a PO and information. | ***Data for risk assessment:*** O2 sats, symptoms, imaging  ***Data for monitoring:*** | PA called patients on a daily basis going through a script of questions about their symptoms. | When the clinical team identified a case of deterioration the patient could be asked to call emergency services or attend ED. | ED attendance, admission, ICU admission, mortality, costs. |
| Pereira Motta [38] | Brazil | Evaluation/ Preprint | Pre-admission | Remote monitoring | SC | Confirmed or suspected cases + non-COVID-19 control group | Not specified | ***Data for risk assessment***: not specified  ***Data for monitoring:*** O2 sats, BPM, temperature, PEF. | Patient asked to input observations into an app as well as keep a paper diary. Measurements are taken twice daily. | The clinical team can monitor patient observations on a dashboard | Indicators of deterioration. |
| Silven [39] | Netherlands | Description of the service/Published article | Pre-admission and step-down | Telemonitoring | SC | Confirmed or suspected cases presenting in the ED or after discharge. Over 18 years, in possession of smartphone + internet, able to communicate through phone. | Patients with mild or moderate symptoms were identified in the ED and onboarded to the service. Patients admitted to COVID-19 ward who were eligible for discharge could also be onboarded. Patients were given a COVID Box containing: PO, BP monitor, thermometer, information. | ***Data for monitoring:*** O2 sats, BP, temperature, HR, RR | In the initial stages of implementation patients kept a record of their observations and these were added manually by th clinical team to their patient chart. In a later stage, patients used an app to record their observations and these were transmitted automatically to the patient’s electronic chart. | A physician of PA carried out daily video consultations to monitor progress. | Admission, mortality |
| Francis [40] | UK | Cohort study/ Preprint | Pre-admission and step-down | Virtual hospital remote assessment | SC | Confirmed or suspected cases presenting in the ED or after discharge. | Patients in the ED or at the point of discharge were assessed for suitability for the service. Patients were triaged based on risk. | ***Data for risk assessment:*** not included in preprint.  ***Data for monitoring:*** O2 sats, BP, temperature | Patients were asked to record their symptoms and observations on paper. | A member of the clinical team called patients regularly to monitor their symptoms and identify any signs of deterioration. | Admission, mortality, predictors of adverse outcomes. |
| Vindrola-Padros [24] | UK | Service evaluation/ Preprint | Pre-admission and step-down | Virtual ward | SC and PC | Confirmed or suspected cases presenting in the ED or after discharge. | The patient was triaged  through emergency telephone numbers, GP practice, or ED. Some were also triaged at the point of discharge. | ***Data for risk assessment:*** not included in preprint.  ***Data for monitoring:*** symptoms, O2 sats, heart rate, temperature and  blood oxygen levels. | The patient was given a pulse oximeter, patient information (including escalation  warning signs and what to do) and a mechanism for recording observations regularly  (app or paper diary). | The patient received regular monitoring calls from staff capturing changes in symptoms. The  sites using apps for patient monitoring triggered alerts if symptoms pointed to deterioration. | Ventilation, mortality, reattendance to ED, admission, ICU admission, call emergency services |
| Wilcock [23] | UK | Prospective study/ Preprint | Pre-admission | Community oximetry monitoring | PC | Confirmed case and not living in a care facility. | After a positive test result, the patient was invited to the service and sent a PO, instructions and diary. | ***Data for risk assessment:*** not included in preprint.  ***Data for monitoring:*** symptoms, O2 sats, degree of breathlessness, Roth score. | Patients recorded their symptoms and O2 sats twice a day on paper. | The patient received regular monitoring calls from staff capturing changes in symptoms. | Deterioration, admission. |
| Clarke [41] | UK | Evaluation/ published article | Pre-admission and step-down | Home oximetry | SC and PC | Confirmed or suspected cases presenting in the ED, primary care or after discharge. | Not specified | Not specified | Not specified | Not specified | ED attendance, admission, mortality |

*Used a pulse oximeter

**Did not use pulse oximetry in the main model described in the article but flagged the launching of a companion programme or incorporating pulse oximetry and escalation based on oxygen saturation at a later date.

***Pulse oximetry added three weeks after implementation

SC=secondary care

PC=primary care

HR= heart rate

RR= respiration rate

PO= pulse oximetry
